# Before leg pain and paraparesis are attributed to vitamin C deficiency alone, comorbidities and cocausalities must be considered and ruled out

**DOI:** 10.51866/lte.7l4

**Published:** 2024-09-18

**Authors:** Sounira Mehri, Josef Finsterer

**Affiliations:** 1 MD, PhD, Neurology Dpt., Neurology ∓ Neurophysiology Center, Vienna, Austria. Email: fifigs1@yahoo.de; 2 PhD, Biochemistry Laboratory, LR12ES05, "Nutrition-Functional Foods and Vascular Health", Faculty of Medicine, Monastir, Tunisia.

**Keywords:** Scurvy, Ascorbic acid, Muscle weakness, Gait disorder

**Dear editor,**

We read with interest the article by Noor Emilia and Said about an 8-year-old boy with autism spectrum disorder (ASD) as well as recurrent fever, fatigue and 3-kg weight loss occurring 3 months prior to admission.^1^ One week before admission, he developed persistent leg pain that worsened with active and passive movements.^1^ Clinical examination revealed cachexia, mild pallor, hyperpigmentation of the gums, multiple hyperpigmented scars on the upper and lower limbs, palpable inguinal lymph nodes, antalgic gait and muscle weakness of the lower limbs (M4).^1^ Scurvy was suspected and diagnosed due to a low serum vitamin C level, and the patient’s symptoms improved after the administration of vitamin C and other vitamins.^1^ The article is impressive, but some points should be discussed.

The first point is that the patient was treated orally not only with vitamin C (300 mg/day) but also with other vitamins. Therefore, it cannot be excluded that the symptoms could be due not only to vitamin C deficiency but also to other vitamin deficiencies. Identifying what kinds of vitamins the patient was given in addition to vitamin C and their dosages is crucial.

The second point is that the serum levels of other vitamins or other serum parameters were not mentioned. In particular, the serum levels of vitamin A, vitamin B1, folic acid, vitamin B6, vitamin B12 and vitamin D must be indicated. Since the patient was on a selective diet, it is conceivable that he developed poly-vitamin deficiency rather than vitamin D deficiency alone. Therefore, the patient’s symptoms could have been caused by a combination of several hypovitaminoses rather than by vitamin C deficiency alone.

The third point is that the cause of ASD was not specified. Whether ASD is inherited or acquired must be determined. For acquired ASD, it is essential to identify whether it is due to vitamin D deficiency, as previously reported.^2^ Additionally, patients with ASD may develop vitamin A deficiency.^3^ An unbalanced diet can also lead to vitamin B12 deficiency and the manifestation of ASD.^4^ It is necessary to review cerebral magnetic resonance images for any structural lesions that could explain ASD.

The fourth point is the discrepancy between the characterisation of the patient’s pain on admission and after vitamin C supplementation. On admission, the pain was described as leg pain, suggesting that the whole leg was affected, while after vitamin C supplementation, it was mentioned as joint pain. It should therefore be clarified whether the patient had joint pain, bone pain, muscle pain, fascial pain, connective tissue pain, skin pain or a combination of these.

The fifth point is that no explanation was given as to what type of infection was responsible for the recurrent fever, the increased inflammatory parameters and the swollen inguinal lymph nodes.

In conclusion, this interesting study has limitations that relativise the results and their interpretations. Addressing these limitations could strengthen the conclusions and reinforce the message of the study. Before leg pain, cachexia, pallor, hyperpigmentation of the gums, multiple hyperpigmented scars on the upper and lower limbs, palpable inguinal lymph nodes, antalgic gait and muscle weakness of the lower limbs are attributed solely to vitamin C deficiency, comorbidities must be considered and carefully excluded.
